# High throughput optical lithography by scanning a massive array of bowtie aperture antennas at near-field

**DOI:** 10.1038/srep16192

**Published:** 2015-11-03

**Authors:** X. Wen, A. Datta, L. M. Traverso, L. Pan, X. Xu, E. E. Moon

**Affiliations:** 1 School of Mechanical Engineering and Birck Nanotechnology Center, Purdue University, West Lafayette, Indiana 47906; 2 Center for Micro- and Nanoscale Research and Fabrication, Hefei National Laboratory for Physical Sciences at the Microscale, University of Science and Technology of China, Hefei, Anhui, 230026, China; 3 Department of Optics and Optical Engineering, the University of Science and Technology of China, Hefei, Anhui 230026, China; 4 Department of Electrical Engineering and Computer Science, Massachusetts Institute of Technology, Cambridge, Massachusetts 02139.

## Abstract

Optical lithography, the enabling process for defining features, has been widely used in semiconductor industry and many other nanotechnology applications. Advances of nanotechnology require developments of high-throughput optical lithography capabilities to overcome the optical diffraction limit and meet the ever-decreasing device dimensions. We report our recent experimental advancements to scale up diffraction unlimited optical lithography in a massive scale using the near field nanolithography capabilities of bowtie apertures. A record number of near-field optical elements, an array of 1,024 bowtie antenna apertures, are simultaneously employed to generate a large number of patterns by carefully controlling their working distances over the entire array using an optical gap metrology system. Our experimental results reiterated the ability of using massively-parallel near-field devices to achieve high-throughput optical nanolithography, which can be promising for many important nanotechnology applications such as computation, data storage, communication, and energy.

For decades, optical nano-patterning has been the key technique to drive the discoveries in many nanotechnology areas. The long-standing target in the optical nano-patterning development is to achieve both high resolution and high throughput at low costs^1^. Particularly, for economically creating periodic structures over a larger area, many effective lithographic techniques have been developed, such as block-copolymer self-assembly lithography, self-assembled nano-particles, and nano-sphere lithography^2^,^3^,^4^,^5^,^6^. Among various types of nano-patterning techniques, near-field scanning optical lithography (NSOL) has attracted much attention^7^,^8^,^9^,^10^,^11^,^12^,^13^,^14^,^15^,^16^,^17^,^18^,^19^,^20^,^21^. NSOL typically scans a probe with nano-apertures as diffraction-unlimited light sources to direct write surface features in the optical near-field. Without the need of pre-patterned templates or photomasks, an arbitrary pattern can be created with NSOL by raster- or vector-scanning the probe over the photoresist-coated substrate. Sub diffraction-limit feature sizes can be obtained using NSOL technique and feature sizes comparable to electron beam lithography on self-assembled monolayers have been reported^14^,^17^. Remarkably high scanning speeds (cm/s) in NSOL have also been demonstrated, albeit with less resolution of around 190 nm^16^ using a solid immersion lens. A major technical challenge in NSOL has been the precise control of the separation or gap distance between the photoresist and the nano-apertures during the lithography process^8^,^11^,^22^. The light emerging from the exit-plane of the nano-aperture is subjected to strong decay and divergence over a distance of tens of nanometers^23^,^24^. Precise control of the gap distance within tens of nanometers is crucial for maintaining both high light intensity and a tight light spot during the lithography process. On the other hand, NSOL does not need to prohibitively scale down the working wavelength to improve process resolution, which makes it inexpensive compared to other state-of-the-art lithography methods. Traditional NSOL uses a single aperture/antenna which poses a limit on the throughput. One way to achieve high patterning throughput is to use a large number of nano-apertures in parallel. Massively-parallel scanning lithography has been demonstrated in scanning probe lithography where large arrays of cantilever probes were used to generate lithographic patterns in the contact mode^15^,^25^,^26^. Dip-pen lithography, a versatile scanning probe lithography method, is capable of producing massively parallel patterns at the 100 nm scale^27^,^28^,^29^. “Snomipede” is a parallel scanning near field lithography method wherein an area as large as 1 mm in size was addressed simultaneously by sixteen probes yielding a minimum feature size of 70 nm^15^. On the other hand, there has been no experimental demonstration of massively parallel near-field mask- less optical lithography of a large number of patterns at diffraction-unlimited resolutions, because of the difficulty in precisely controlling the gap distance between a nano-aperture array and the photoresist surface over a large area.

In this paper, we report our recent experimental advances in achieving massively parallel near-field maskless optical lithography with diffraction-unlimited resolutions. Our approach was accomplished by integrating a bowtie-aperture-based near-field parallel scanning lithography system with an optical metrology system, named interferometric-spatial-phase-imaging (ISPI), to detect and actively control the near-field gap distance. The ISPI system uses a different wavelength of laser light that is insensitive to the photoresist to avoid interfering with the lithography system. Measured pattern line-width as narrow as 19 nm in the deep sub-wavelength scale was achieved by precisely controlling the working distance of the nano-apertures. Moreover, up to 1,024 nano-patterns were created in parallel. In principal, there is no fundamental limit to further scale up the parallel patterning throughput by the use of an even larger number of nano-antennas.

Figure 1a shows the schematic of the parallel NSOL system, which consists of two optical systems: the lithography system and the optical gap detection and alignment system which includes an interferometric method for coarse alignment as the first step that is described elsewhere^10^ and an interferometric-spatial-phase-imaging (ISPI) setup for subsequent fine alignment and gap control. In the lithography system, a frequency-tripled diode-pumped solid state UV laser (wavelength = 355 nm) is utilized as the light source for exposing photoresist. An optical mask carrying light focusing elements, i.e. an array of bowtie aperture antennas, is mounted on a multi-axis piezoelectric stage and leveled with respect to a photoresist-covered flat substrate mounted onto a scanning stage. Figure 1b shows a schematic sketch of the mask, which is made in a piece of 0.5′′ × 0.5′′ quartz substrate. A square island of 150 μm side length is made at the center of the quartz substrate. A 70-nm thick chromium layer is coated onto top of the island, in which bowtie nano-apertures with a nominal outline dimension of 190 nm by 190 nm, designed to achieve the highest transmission^23^,^30^, were milled using focused ion beam (FIB). The SEM images of the mask and an array of bowtie apertures are shown in Fig. 1c,d. The pitch of the bowtie aperture array is chosen to be 2 μm, which is significantly larger than the diffraction limit and the surface plasmons wave decay distance. This arrangement allows us to individually address each bowtie aperture antenna using diffraction-limited optics and avoid potential interference between neighboring apertures. On both sides of the island, a series of two-dimensional chirped gratings are made for ISPI gap detection as shown in Fig. 1b, which will be described next.

For ISPI gap detection, a red laser beam (660-nm wavelength, which is insensitive to the photoresist) incidents at an oblique angle onto the ISPI gratings and diffracts into different angles due to the chirped period of the gratings. A portion of the diffracted beam reflects back from the substrate surface and re-diffracts on the gratings, creating a set of interference fringes which is captured by a camera. The number and positions of the fringes are sensitive to the distance between the mask and substrate, based on which the gap distance is computed. More details of the ISPI technique were given elsewhere^31^,^32^,^33^,^34^.

Before lithography, Shipley S1805 photoresist was spun on a quartz substrate at 4500 rpm, giving a thickness of 400 nm. The mask and the resist substrate were held with 5-axis degree-of-freedom, all controlled by piezoelectric stages. The resist substrate can scan in x, y and z directions with a positioning resolution of 0.4 nm. The stage can also adjust its tip-tilt angle to obtain a uniform gap between the mask and resist substrate. At the initial stage of lithography, the working gap between the resist substrate and the mask is calibrated by first bringing the substrate into light contact with the island on the mask and then lifting the mask to a desired working distance. Since the surface roughness of the undeveloped photoresist is less than 1.5 nm, it does not have a significant effect on the ISPI readings. During lithography, the mask is illuminated by the UV exposure laser beam. By modulating the laser beam according to the motion of the resist substrate in the x-y plane vector scan, an arbitrary pattern can be produced.

## Results and Discussion

The control of the gap between the mask and the substrate contributes greatly to the quality of the lithography result. When the working gap is larger than tens of nanometers, the transmitted optical energy will be too low to expose the photoresist at high throughput and the optical hot spot will diverge significantly, leading to a poor lithography resolution. If the aperture mask and the photoresist are too close, i.e., less than a few nm, the interfacial forces start to play a role in mechanical alignment and scanning processes. When the aperture and the photoresist are in contact, the adhesion and friction may cause unfavorable hysteresis in scanning trajectories, photoresist deformations, and even material damages. The ideal working distance is to bring the mask as close to the substrate as possible, but avoid any contact. Besides the gap distance, the scanning speed and exposure dose are also important parameters which contribute significantly to the quality of the lithographic patterns. For a particular gap distance, increase of scanning speed means less exposure time at a given location which can cause shallower or even no lithography results. Conversely, for a particular scanning speed, increase of exposure dose makes the lithographic lines thicker due to more energy being deposited. It was found experimentally that the required exposure dose increases roughly linearly with the scanning speed.

Any possible imperfections of the substrate and the mask will also have some influences on the lithography quality. It was found that the roughness of the photoresist lies within 1.5 nm and the specified flatness of the quartz substrates that were used for the mask and the substrate is λ/20 (λ = 633 nm) = 37 nm over a 0.5′′ x 0.5′′ area, which translates to a flatness better than 1 nm over the 150 μm x 150 μm island where the bowtie apertures are fabricated.

**Figure 2:**
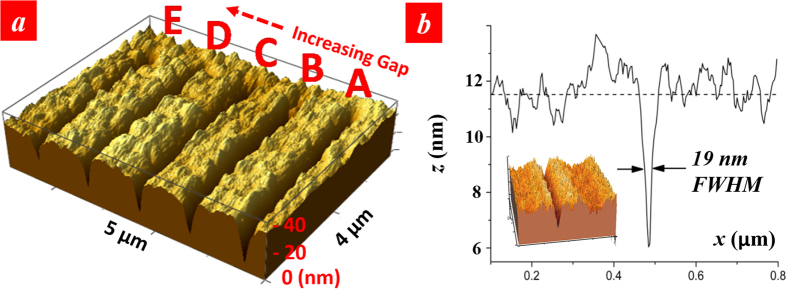
Working distance optimization. (**a**) AFM image of lines produced with various mask-substrate distance: A: 0 nm, B: 5 nm, C: 10 nm, D: 15 nm, E: 20 nm. (**b**) Cross section of a line produced with 10-nm mask-substrate distance.

As shown in Fig. 2a, we precisely measured and controlled the gap distance at nanoscale and perform lithography at different gap distances varying from 0 to 20 nm while keeping the exposure dose and scan speed unchanged, at 1.1 μJ/cm^2^ and 0.7 μm/s, respectively. The exposure dose is not a strong function of the gap distance within this distance range. The depth of the lines decreases with the increasing distance. All the AFM topographical images in this work have been taken in the contact mode with a Bruker ORC8-10 AFM probe with a nominal tip radius of 15 nm. At a gap distance of 10 nm, the minimum line-width of 19 nm measured at full-width-high-maximum (FWHM) was obtained by optimizing the exposure dose (Fig. 2b) to be 0.67 μJ/cm^2^ and 0.5 μm/s, respectively. Since the feature size is similar to the dimension of the tip radius, the effect of convolution of the tip shape with the feature size could affect the measured FWHM value. Deconvolution of the measured line profile with the geometric model of the tip shows a FWHM value of 25 nm. We believe in this case the transmitted dose through the bowtie aperture is just above the photoresist exposure threshold.

**Figure 3:**
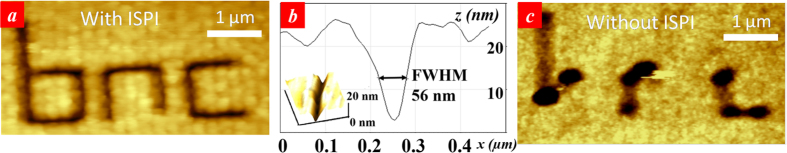
(**a**) AFM image of lithography result of letters “bnc” with alignment using ISPI technique, (**b**) the cross sectional profile of the line pattern. (**c**) A typical lithography results of letters “bnc” without using ISPI alignment.

During parallel lithography operation, we consistently maintain a parallelism between the mask and substrate better than 30 μrad^32^. This ensures that the mask operates at an optimized working distance with a variation less than 5 nm across the entire aperture array of 150 μm by 150 μm in size. A larger variation in working distance caused by uncompensated tilt angles will induce pattern distortions and lead to poor pattern uniformity over the array due to the near-field nature of the optical field emerging from the nano-apertures. Figure 3a shows the lithography results of letters “bnc” when the tilt is controlled below 30 μrad using the ISPI technique. The FWHM of the line is around 56 nm as shown in Fig. 3b. Figure 3c shows a comparison lithography results where the tilt angle was not adjusted using the ISPI technique. It is clearly seen that, in the absence of tilt angle control, variation in working distances caused severe hysteresis in scanning trajectories and pattern distortions.

**Figure 4:**
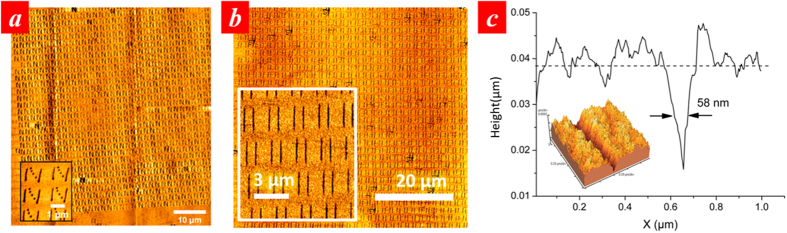
Massive parallel lithography results produced by scanning 1,024 bowtie aperture. (**a**) at a linear speed of 1 μm/s, patterning an array of letter “N” and (**b**) at a linear speed of 10 μm/s producing an array of lines. (**c**) A cross sectional profile of the line pattern.

In parallel lithography experiments, we uniformly illuminated the aperture array although they can be individually addressed to generate fully arbitrary patterns by using a micro-scale optical projection system powered by spatial light modulators, such as digital micro-mirror device (DMD). Figure 4 shows the parallel lithography results using a 1,024-bowtie aperture array achieved at an optimized gap distance and light power level. Figure 4a shows an array of “N” shape patterns exposed, consisting of two lines and dots in the middle as seen in the inset of Fig. 4a. The mean linewidth of the lines of the “N” shape shown in the inset is 67 nm with a standard deviation of 8 nm. This variation in the linewidth is most likely caused by the variation in the fabrication of each of the bowtie apertures. Characterization of the dimensions of the bowtie apertures from their SEM images showed a variation of about 7 nm in the gap distance between the two arms of the bowtie apertures and the outline dimension. The result in Fig. 4a was obtained using an exposure dose of 1.57 μJ/cm^2^ and a linear scanning speed of 1 μm/s. This higher laser fluence was used to ensure patterns are produced by all the antennas since there is variation between the antennas made using FIB. We also tested using higher scan speeds. With a faster scan speed, the exposure light power intensity needed to be increased accordingly. Figure 4b shows the lithography result obtained at a scan speed of 10 μm/s, where the laser fluence was increased by approximate 10 times to 17 μJ/cm^2^. Laser heating might increase the temperature of the apertures, but the expansion of the metal film caused by a temperature rise of even 100 °C is estimated less than 0.1 nm, hence the performance of the individual bowtie apertures is not likely to be affected. The FWHM line-width of one of the lines is shown in Fig. 4c. Detailed characterization of the linewidth by zoomed-in AFM scans over the whole range of the patterned area show that the mean linewidth is 73 nm with a standard deviation of 15 nm.

In summary, we demonstrated massively parallel near-field scanning lithography utilizing a large array of bowtie antenna apertures, with a resulting linewidth of about 70 nm. The capability of massively parallel near-field nano-lithography was enabled by the precise control of the working distance of the apertures. Uniformity of the bowtie aperture fabrication can be the key for achieving uniform lithography patterns with higher resolution. This approach has the potential to renovate the single-aperture NSOL to achieve a much higher throughput. It offers a promising optical nanolithography strategy for nano manufacturing needs.

## Additional Information

**How to cite this article**: Wen, X. *et al.* High throughput optical lithography by scanning a massive array of bowtie aperture antennas at near-field. *Sci. Rep.* **5**, 16192; doi: 10.1038/srep16192 (2015).

## Figures and Tables

**Figure 1:**
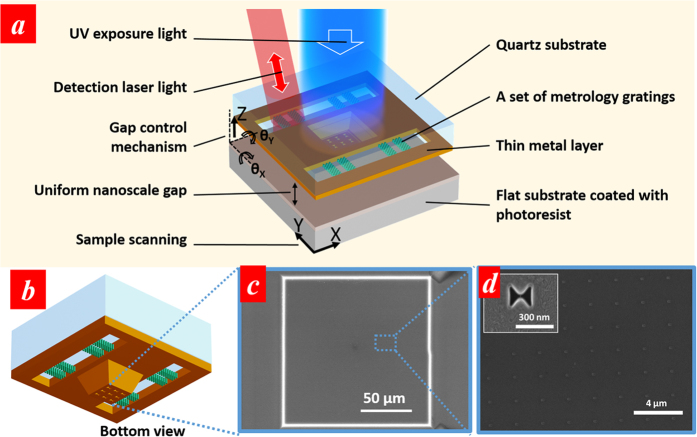
(**a**) Schematic of the parallel near-field scanning optical lithography (NSOL) system. (**b**) Schematic of NSOL mask with a flat island of protrusion. (**c**) SEM image of an island milled with an array of bowtie apertures. (**d**) Zoom-in SEM image of the bowtie aperture array. Inset is the SEM image of an individual bowtie aperture. (**a,b**) were drawn by X.W.

## References

[b1] Xie Z. *et al.* Plasmonic Nanolithography: A Review. Plasmonics 6, 565–580 (2011).

[b2] Hulteen J. C. & Van Duyne R. P. Nanosphere lithography: a materials general fabrication process for periodic particle array surfaces. J. Vac. Sci. Technol. A 13, 1553–1558 (1995).

[b3] Haginoya C., Ishibashi M. & Koike K. Nanostructure array fabrication with a size-controllable natural lithography. Appl. Phys. Lett. 71, 2934–2936 (1997).

[b4] Cheng J. Y., Ross C., Thomas E., Smith H. I. & Vancso G. Fabrication of nanostructures with long-range order using block copolymer lithography. Appl. Phys. Lett. 81, 3657–3659 (2002).

[b5] Park M., Harrison C., Chaikin P. M., Register R. A. & Adamson D. H. Block copolymer lithography: periodic arrays of ~1011 holes in 1 square centimeter. Science 276, 1401–1404 (1997).

[b6] Guo W. *et al.* Near-field laser parallel nanofabrication of arbitrary-shaped patterns. Appl. Phys. Lett. 90, 243101 (2007).

[b7] Pan L. *et al.* Maskless plasmonic lithography at 22 nm resolution. Sci. Rep. 1, 175, doi: 10.1038/srep00175 (2011). 22355690 PMC3240963

[b8] Kim T. *et al.* High-speed plasmonic nanolithography with a solid immersion lens-based plasmonic optical head. Appl. Phys. Lett. 101, 161109 (2012).

[b9] Wang L., Uppuluri S. M., Jin E. X. & Xu X. Nanolithography using high transmission nanoscale bowtie apertures. Nano Lett. 6, 361–364 (2006). 16522023 10.1021/nl052371p

[b10] Uppuluri S., Kinzel E. C., Li Y. & Xu X. Parallel optical nanolithography using nanoscale bowtie aperture array. Opt. Express 18, 7369–7375 (2010). 20389758 10.1364/OE.18.007369

[b11] Murphy-DuBay N., Wang L. & Xu X. Nanolithography using high transmission nanoscale ridge aperture probe. Appl. Phys. A 93, 881–884 (2008).

[b12] Wen X., Traverso L. M., Srisungsitthisunti P., Xu X. & Moon E. E. Optical nanolithography with λ/15 resolution using bowtie aperture array. Appl. Phys. A 117, 307–311 (2014).

[b13] Sun S., Chong K. S. & Leggett G. J. Nanoscale molecular patterns fabricated by using scanning near-field optical lithography. J. Am. Chem. Soc. 124, 2414–2415 (2002). 11890771 10.1021/ja017673h

[b14] Sun S. & Leggett G. J. Matching the resolution of electron beam lithography by scanning near-field photolithography. Nano Lett. 4, 1381–1384 (2004).

[b15] Haq E. U. *et al.* Parallel Scanning Near-Field Photolithography: The Snomipede. Nano Lett. 10, 4375–4380 (2010). 20945880 10.1021/nl1018782

[b16] Ghislain L. P. *et al.* Near-field photolithography with a solid immersion lens. Appl. Phys. Lett. 74, 501–503 (1999).

[b17] Montague M. *et al.* Fabrication of biomolecular nanostructures by scanning near-field photolithography of oligo (ethylene glycol)-terminated self-assembled monolayers. Langmuir 23, 7328–7337 (2007). 17511486 10.1021/la070196h

[b18] Liao X. *et al.* Desktop nanofabrication with massively multiplexed beam pen lithography. Nat. Commun. 4, 2103, doi: 10.1038/ncomms3103 (2013). 23868336 PMC3807695

[b19] Krausch G. & Mlynek J. Surface modification in the optical near field. Microelectron. Eng. 32, 219–228 (1996).

[b20] Wegscheider S., Kirsch A., Mlynek J. & Krausch G. Scanning near-field optical lithography. Thin solid films 264, 264–267 (1995).

[b21] Sun S. & Leggett G. J. Generation of nanostructures by scanning near-field photolithography of self-assembled monolayers and wet chemical etching. Nano Lett. 2, 1223–1227 (2002).

[b22] Srituravanich W. *et al.* Flying plasmonic lens in the near field for high-speed nanolithography. Nat. Nanotechnol. 3, 733–737 (2008). 19057593 10.1038/nnano.2008.303

[b23] Jin E. X. & Xu X. Finitte-Difference Time-Domain Studies on Optical Transmission through Planar Nano-Apertures in a Metal Film. Jpn. J. Appl. Phys. 43, 407–417 (2004).

[b24] Srisungsitthisunti P., Ersoy O. K. & Xu X. Improving near-field confinement of a bowtie aperture using surface plasmon polaritons. Appl. Phys. Lett. 98, 223106–223103 (2011).

[b25] Liu C. Parallel scanning probe arrays: their applications. Mater. Today 11, 22–29 (2008).

[b26] Minne S., Manalis S., Atalar A. & Quate C. Independent parallel lithography using the atomic force microscope. J. Vac. Sci. Technol. B 14, 2456–2461 (1996).

[b27] Piner R. D., Zhu J., Xu F., Hong S. & Mirkin C. A. “Dip-pen” nanolithography. Science 283, 661–663 (1999). 9924019 10.1126/science.283.5402.661

[b28] Salaita K. *et al.* Sub‐100 nm, Centimeter‐Scale, Parallel Dip‐Pen Nanolithography. Small 1, 940–945 (2005). 17193372 10.1002/smll.200500202

[b29] Salaita K. *et al.* Massively Parallel Dip–Pen Nanolithography with 55 000‐Pen Two‐Dimensional Arrays. Angew. Chem. 118, 7378–7381 (2006). 10.1002/anie.200603142 17001599

[b30] Xu X., Jin E., Uppuluri S. & Wang L. Concentrating light into nanometer domain using nanoscale ridge apertures and its application in laser-based nanomanufacturing. Paper presented at Eighth International Conference on Laser Ablation, 11–16 September 2005, Banff, Canada. Published in *J. Phys. Conf. Ser*. 59, 273-278, IOP Publishing (2007), doi: 10.1088/1742-6596/59/1/057.

[b31] Moon E. E., Chen L., Everett P. N., Mondol M. K. & Smith H. I. Interferometric-spatial-phase imaging for six-axis mask control. J. Vac. Sci. Technol. B 21, 3112–3115 (2003).

[b32] Wen X., Traverso L. M., Srisungsitthisunti P., Xu X. & Moon E. E. High precision dynamic alignment and gap control for optical near-field nanolithography. J. Vac. Sci. Technol. B 31, 041601–041605 (2013).

[b33] Srisungsitthisunti P. *et al.* Nanometer-level alignment using interferometric-spatial-phase-imaging (ISPI) during silicon nanowire growth. In Instrumentation, Metrology, and Standards for Nanomanufacturing IV, edited by Michael T.Postek & John A Allgair , Proc. SPIE 7767 (SPIE, Bellingham, WA, 2010) (August 24, 2010), doi: 10.1117/12.860581.

[b34] Moon E. *Interferometric-spatial-phase imaging for sub-nanometer three dimensional positioning* PhD Thesis, Massachusetts Institute of Technology (2004).

